# A Case of Chronic Lymphocytic Leukemia Coinciding With COVID-19

**DOI:** 10.7759/cureus.42522

**Published:** 2023-07-26

**Authors:** Mohammed A Jeraiby

**Affiliations:** 1 Department of Medical Biochemistry, Faculty of Medicine, Jazan University, Jazan, SAU

**Keywords:** covid-19, lymphocytosis, lymphocytosis chronic lymphocytic leukemia, leukemia, blood morphology

## Abstract

Comorbidities including leukemia are risk factors in coronavirus disease 2019 (COVID-19) patients for high morbidity and mortality. The severity of the disease is usually correlated with lymphopenia. On the other hand, we came across a case of marked absolute lymphocytosis in a COVID-19 patient, which further escalated five-fold during his hospital stay. Subsequently, the diagnosis of chronic lymphocytic leukemia (CLL) was made following positive cell surface markers for CD19+, CD5+, and CD20+ (dim), in the presence of restricted immunoglobulin light chain of lambda type on flow cytometry. Numerous cases are available in the literature of COVID-19 among established CLL patients. However, we are mentioning here the second case where the diagnosis of CLL was established accidentally during the work-up for lymphocytosis in COVID-19 infection.

## Introduction

Clusters of pneumonia of unknown etiology were presented at the end of 2019 in Wuhan, China. The novel severe acute respiratory syndrome coronavirus 2 (SARS-CoV-2) belonging to the beta-coronavirus family turns out to be the causative agent of coronavirus disease 2019 (COVID-19) [1]. Typically, clinical presentations of the infection are fever, headache, and fatigue, accompanied by respiratory symptoms like cough and dyspnea, and other systemic involvements. However, a considerably higher death rate was observed in serious manifestations of the disease. Male gender, advanced age, and comorbidities including cancer are identified as independent risk factors for high mortality among COVID-19 patients. Additionally, immunosuppressive therapeutic interventions in conjunction with accompanying immunodeficiency in hematological malignancies can potentiate the risk of critical illness in COVID-19 infections [2].

Chronic lymphocytic leukemia (CLL) is a type of non‐Hodgkin lymphoma associated with severe immunodeficiency [3]. Due to the disease of the elderly and accompanying severe immune disorders, patients with CLL may be at particular risk for COVID-19 and its associated complications [4,5]. In addition, symptomatic COVID-19 among previously diagnosed CLL patients was reported frequently with a prevalence of approximately 1% [6]. However, only one case of COVID-19 infection has been reported so far with no prior diagnosis of CLL [7]. Here, we describe a case of COVID-19 with marked lymphocytosis, an incidental finding on complete blood counts, and a peripheral blood picture, which was later diagnosed as CLL.

## Case presentation

A 61-year-old man presented with a fever, headache, malaise, and cough for five days. He re­ported weight loss of around 10 kilograms in the last month with no appreciable change in his appetite. He was also passing loose stools around 10 episodes/day for the last three days with rectal bleeding for two days. The patient gave a history of hemorrhoidectomy and had postoperative anal incontinence. There is no history of constipation or abdominal pain or any long-term medication. No family history of malignancies, especially colon cancer, or symptoms of anemia.

On physical examination, he was stable with high-grade fever (39.6 C) and mild dyspnea with oxygen saturation of 99% by pulse oximetry on room air. The abdomen was soft with mild distension. The liver and spleen were not enlarged, and no palpable mass was noticed. There was no lymphadenopathy. Per a rectal examination, a hemorrhoid was noted at 3 o’clock with no active bleeding. He had decreased bilateral air entry on auscultation. The remainder of the examination was unremarkable.

Chest X-ray showed patchy consolidation opacities. Laboratory findings were severe leukocytosis with absolute lymphocytosis at the time of hospital admission which further increased to 3-to 5-fold after two weeks. C-reactive protein (CRP) and erythrocyte sedimentation rate (ESR) were mildly elevated. Slightly high levels of aspartate aminotransferase and alanine aminotransferase were also noted. A screen for metabolic liver diseases was unyielding, as were tests for viral hepatitis. Arterial blood gas, serum electrolytes, and renal function tests were within normal limits. The urine dipstick test was negative for protein and blood (Table 1).

**Table 1:** Results of patient investigation Laboratory analysis screening with normal range. WBC: absolute count of leukocyte, RBC: absolute count of red cells, HGB: Hemoglobin, CRP: c-reactive protein, MCV: mean corpuscular volume, MCH: mean corpuscular hemoglobin, MCHC: mean corpuscular hemoglobin concentration, RDW: red cell distribution width, HCT, Hematocrite, LDH: Lactate dehydrogenase, ALP: alkaline Phosphatase, AST:  Aspartate aminotransferase, ALT: alanine aminotransferase.

Variable (unit)	Result		Normal range
	Admission	2 weeks after admission	
WBC (x 10^9^/L)	24.52	83.03	4.5 – 10
RBC(x 10^12^/L)	5.3	4.81	4 – 6.1
HGB (g/dL)	15.6	14.1	11.5 – 16.5
HCT (%)	46.3	45.1	40 – 55
MCV (fL)	87.4	93.8	76 – 96
MCH (pg)	29.4	29.3	27 – 32
MCHC (g/dL)	33.7	31.3	30 – 35
Platelets (x 10^9^/L)	250	320	150 – 400
Neutrophil %	33.9	13.4	40 – 75
Absolute Neutrophil count	8.32	11.08	2.5 – 7.5
Lymphocyte %	52.4	81.6	20 – 45
Absolute Lymphocyte count	12.84	67.74	1.5 – 3.5
Absolute Monocyte count	3.26	3.59	0.04 – 0.4
Sodium (mmol/L)	139	136	135 –153
Potassium (mmol/L)	4.28	5.24	3.5 – 5.3
BUN (mmol/L)	4.6	8.1	2.6 – 6.4
Creatinine (umol/L)	36	67	50 –115
Bilirubin (total) (umol/L)	10.2	12.3	0 – 17
ALP (U/L)	53	52	50 – 137
ALT (U/L)	104.7	34.1	10 – 50
AST (U/L)	54.2	19.3	15 – 37
Total protein (G/L)	50.1	54.2	64 – 82
Albumin (G/L)	32.1	38.1	34 – 50
CRP (mg/dl)	6.4	0.2	0 – 0.3
ESR (mm/Hour)	43	22	2 –20
LDH (U/L)	276	272	100 – 190
Ferritin (Ug/L)	595.5	1032	30 – 400

In light of the patient’s history and radiological evaluation, COVID-19 infection was suspected and the diagnosis was confirmed by RT-PCR (real-time reverse transcriptase polymerase chain reaction).

**Figure 1:**
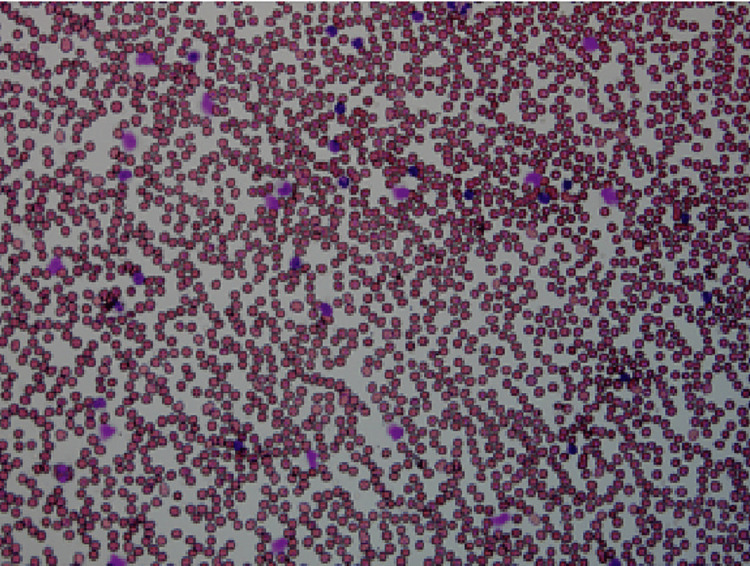
A peripheral blood smear The image shows lymphocytosis, mature lymphocytes with smudge cells.

Further, a thorough work-up on his lymphocytosis was undertaken. Peripheral blood film showed leukocytosis mainly consisting of lymphocytes with smudge cells in the background. Lymphocytosis was noted with normocytic normochromic red blood cells. Platelets were adequate in the count and normal in morphology (Figure 1).

**Figure 2:**
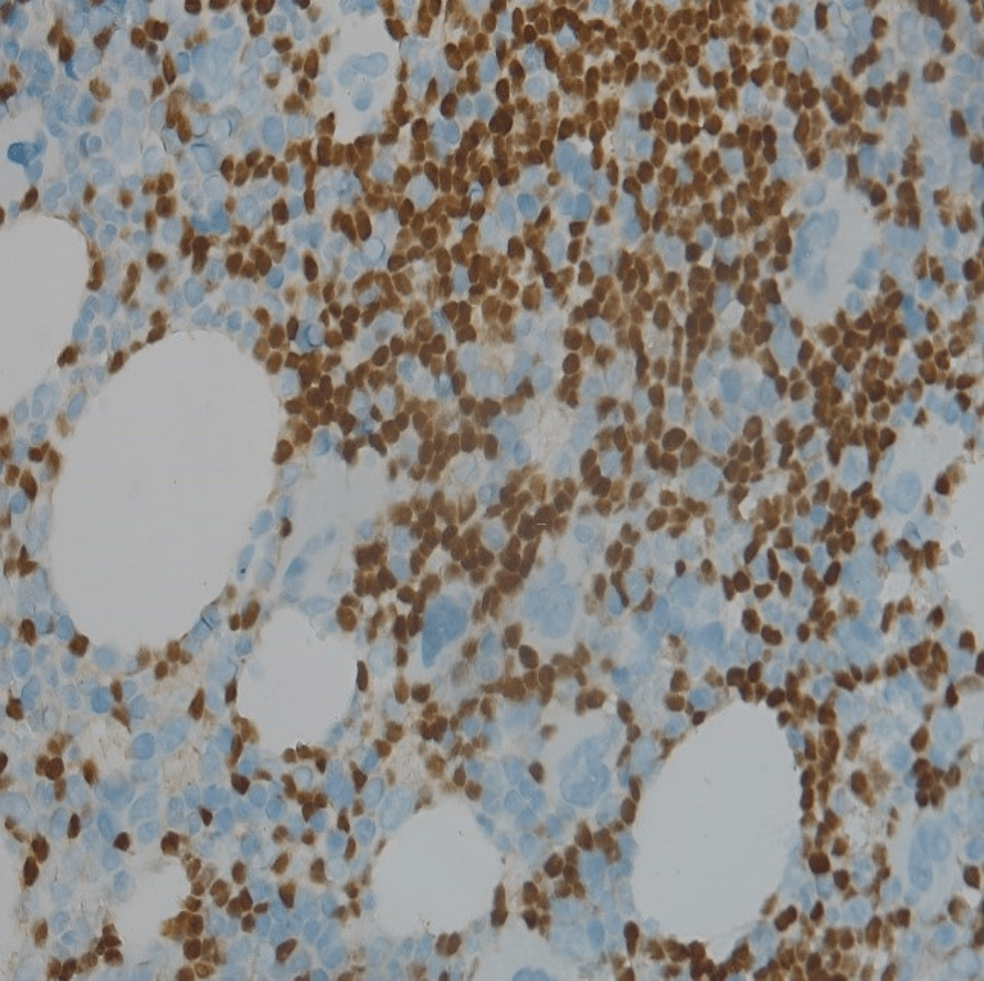
Bone marrow biopsy highlighted by PAX-5 immunohistochemistry stain The image shows scattered lymphocytes spread interstitially highlighted by PAX-5 immunohistochemistry stain (PAX 5 is a nuclear transcription factor that plays a major role in B-cell differentiation and proliferation).

Bone marrow biopsies were stained by PAX 5 immunohistochemistry revealing scattered lymphocytes that spread interstitially (Figure 2).

**Figure 3:**
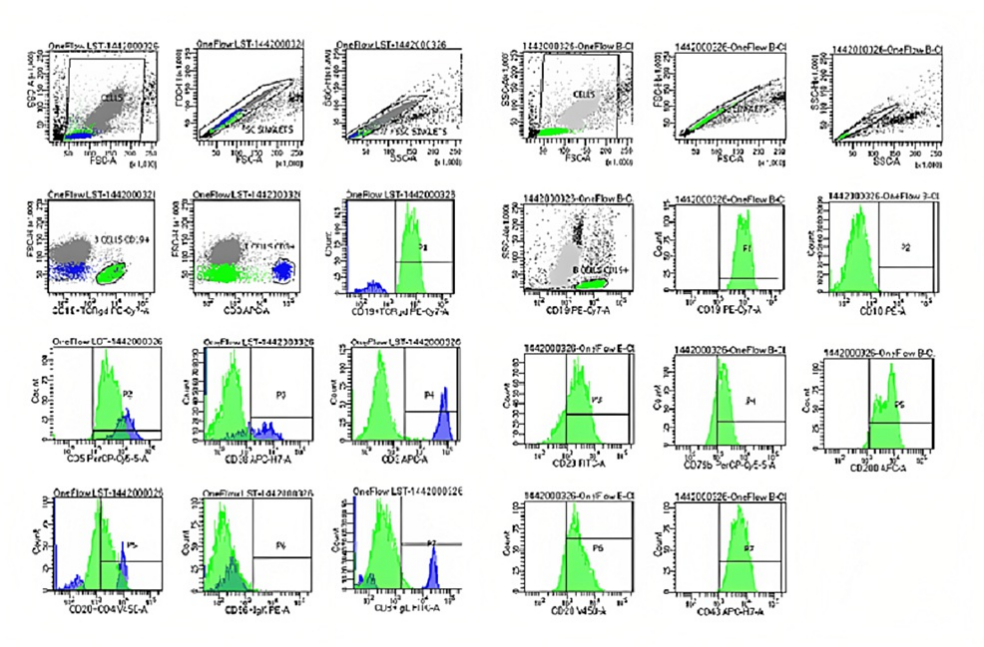
Flow cytometry result images Flow cytometry confirmed this patient has chronic lymphocytic leukemia (CLL).

Flow cytometry was done on a peripheral blood sample using a BD FACS Canto™ II cytometer (Beckton Dickinson Biosciences, USA) (Figure 3). It showed around 11.5% of cells positive for CD45 (in the lymphocyte area). These cells were positive for CD19+, CD5+, CD20+ (dim), CD23+, CD43+, and CD200+ with restricted lambda light chain and negative for CD10+.

Taken together with the findings of flow cytometry and peripheral blood morphology, a diagnosis of CLL was confirmed. The patient was discharged after two weeks to follow up at Hematology/Oncology Clinic.

## Discussion

CLL is the most common leukemia, worldwide, with extreme phenotypic presentation and met with considerable challenges for appropriate diagnosis. Most patients with CLL are diagnosed accidentally while going through their routine blood investigation which reflects absolute lymphocytosis, or during evaluation for enlarged lymph nodes [8]. The Patients having no symptoms at the time of diagnosis carry a good prognosis [9]. Our patient initially presented with moderate COVID-19 symptoms [10]; concurrently, he developed a high lymphocyte count including smudge cells on a peripheral blood smear. His diagnosis was confirmed, per the criteria laid down by Hallek et al., on immunophenotype evaluation by flow cytometry that revealed typical phenotype of CLL with CD19+, CD5+, CD20+ (dim), and restricted lambda light chain immunoglobulins [11].

Further, lymphopenia is a typical finding in COVID-19, among patients with no underlying disease or comorbidities other than CLL, which inversely correlate with the severity of infection, and both T-and B-cells depletion was observed [12,13]. In addition to the reduced counts, T-cells are functionally impaired due to cytokine storm, especially in the serious outcome [12]. On the contrary, we observed a five-fold increase in lymphocyte count, from 12.48 to 67.74 in two weeks after admission. Though the reason behind such phenomenon is obscure, two previous reports had mentioned a threefold increase in lymphocyte count from baseline among therapy-naïve CLL patients having concomitant COVID-19 infection and termed it as “COVID-19-Induced Lymphocytosis” (CIL) [14,15]. Unfortunately, four (with a mean age of 80 years) among the five CLL patients, having no active treatment, described priorly to CIL, succumbed to their illness. Recently, it was found in a multicentric study that CLL patients not on anti-leukemic therapy and with an age of more than 65 years are at higher risk for mortality during COVID-19 infection [6]. In the same study, advanced age appears to be the most important risk factor as 74% of CLL patients having serious COVID-19 were more than 65 years age [6]. It might be possible that our case had a good outcome due to his age, however, the underlying mechanism for CIL among CLL patients should be discerned.

The patient described here is the second case, only after a report by Ali et al., which presented having symptoms of COVID-19 with no prior diagnosis of CLL [7]. Despite the common presentation, there are certain points that make our case different from those previously mentioned. Firstly, the patient reported by Ali et al. was younger, 49 compared to 61 years old, than our case [7]. Secondly, they had encountered a two-fold rise in CIL over four weeks; however, in the present case, it is a fivefold elevation after two weeks. Unlike our patient, five previously reported cases of CIL were established cases of CLL [14,15]. In the absence of any prior evidence of hematological abnormalities in our patient, it is difficult to ascertain whether he had undiagnosed CLL or the present COVID-19 infection had precipitated lymphocytosis in a previously unrecognized monoclonal B-cell lymphocytosis (MBL). There is substantial literature supporting that common community-acquired infections are a risk for the future development of CLL. However, such a phenomenon is a multi-step process, and hypogammaglobinemia as well as MBL, were observed in asymptomatic individuals several years prior to the development of CLL [16]. Therefore, it is possible that our case having undiagnosed MBL might have progressed to CLL during COVID-19 infection, however, we don’t have any direct evidence to support such a conclusion. Patients with CLL are at a higher risk of infection due to defects in the immune system (mostly humoral and cellular). They have hypogammaglobulinemia, T-cell subset abnormalities, and deficiencies. Low immunity makes these patients susceptible to infections, most commonly respiratory infections, CLL patients with inadequate immunity are more vulnerable to COVID-19, which is largely a droplet virus [17].

## Conclusions

This case report describes the second case of COVID-19 complicated with marked absolute lymphocytosis. Subsequently, the diagnosis of chronic lymphocytic leukemia (CLL) was made. Numerous cases of COVID-19 among established CLL patients are reported in the literature. However, we are mentioning here the second case where the diagnosis of CLL was established accidentally during the work-up for lymphocytosis in COVID-19 infection. These data suggest that the subgroup of CLL patients admitted with COVID-19, regardless of disease phase or treatment status, are at high risk of death.
